# Bibliography

**Published:** 1907-04-15

**Authors:** 


### BIBLIOGRAPHY.

ANATOMICAL NOMENCLATURE. This is an octavo volume of about 100 pages, edited by Professor L. F. Barker, of Johns Hopkins University, published by Blackstone's, in which he has put into accessible form for American students, the result of the B. N. A., which is the Basle Anatomical Nomenclature Commission. The idea is to reduce to a systematic and condensed vocabulary the names heretofore given to all the macroscopic anatomical organs and tissues of the body. It leaves out all duplicate or synonymous terms and selects the most significant and appropriate name and puts it into good latin. On one page of the book is printed the latin names of a certain region and directly opposite the English translation of the same. The hope of the commission however is that all writers and teachers shall use only the latin names. The third molar tooth is "Dens Seratinus," or in English the late tooth; the biscupid teeth are "Dentes Prae molares," the inferior maxillary nerve is the nervi Mandibularis," dentin is "Substantia Eburnae." Enamel is Substantia "Adamantina," etc, etc. There is no doubt but this work will in time result in more accurate writing and reference in speaking of anatomical relations, but the technical applications will keep many of the old terms in use for generations to come. The work is well printed and should be in the library of everyone who writes , or is interested in anatomical research of any kind.

AMERICAN TEXT BOOK OF PROSTHETIC DENTISTRY. This is the third edition of this noted text book, and is just issued from the press of Lea Brothers & Co. This edition is edited by Charles R. Turner, Professor of Mechanical Dentistry in the University of Pennsylvania. It is a large octavo volume of nearly nine-hundred pages, and is undoubtedly the best work of its kind ever published. The editor and publishers are both to be congratulated on the manner of treatment and style of presentation. The work has not only been revised, but largely rewritten and is up-to-date so far as it is practicable to make a book on such a subject. The matter is contributed by twelve different authors, each well known and fully qualified to present his portion with authority. As a rule we are not partial to text books made on this plan, as they are apt to contain repetitions and sometimes even conflicts, which perplex the student; so far as we have been able to examine this work we must commend the editor for his wisdom and vigilance in overcoming the cyclopedic tendencies of such a volume. The new chapters on, "Bite Taking," "Plate Retention," and "Selection of Teeth," by the editor are very well done. The article on Celuloid has been left out because as the editor puts it the material is so little used that it was not worth while. This we regard as a mistake as this material is one of the useful and artistic bases that should be used more than it is. The chapters on crown and bridge work are not up-to-date, and could be improved; this was not done perhaps for the same reason that orthodontia is not treated in the book, because it has developed into a specialty by itself.

The chapter on the Laboratory is much more complete than has ever before been presented in any text book, and will give many valuable suggestions as to the proper equipement for an up-to-date laboratory. Many practitioners could read this chapter, and even study it with profit. It is remarkable what an indifferent portion of the usual dental office the laboratory occupies, any place seems to be good enough, when in truth,it should be a large well lighted room,and equipped with modern and efficient appliances for all kinds of modern prosthetic technics. The entire book is well illustrated which of course adds much to its value; it is also well printed. We regard this as one of the most important contributions to our literature of recent years, and we should be glad if some one would devise a scheme for getting it into the hands ot all practitioners, as well as the students in the colleges. The cost of the book is six dollars, which seems large to students in college and also to practitioners; but this price is really insignificant when the cost of its production is considered, and its value to any one who will take the time to read it. We should much rather do without an electric engine or a cabinet, or many other articles of the modern outfit which cost many times as much. In fact we never could understand how dentists could be so indifferent to books, they are absolutely essential to progress and systematic practice. We sincerely trust this book may have a large sale, as it certainly deserves it.

